# Predictive codes of interoception, emotion, and the self

**DOI:** 10.3389/fpsyg.2014.00189

**Published:** 2014-03-04

**Authors:** Alejandra Sel

**Affiliations:** Department of Psychology, Royal Holloway University of LondonEgham, Surrey, UK

**Keywords:** interoception, emotion, selfhood, predictive coding, anterior insular cortex

Interoception is the ability to perceive and integrate physiological signals from within the body. It is closely related to the autonomic system and is a key component in the generation of affective states and abstract representations of the self (Critchley et al., 2004; Ainley and Tsakiris, 2013). Seth proposes a predictive coding (PC) model of interoception that involves a free-energy based explanation of emotion awareness and selfhood. In this model, emotions, and in turn the sense of self, rely on predictions of the causes of interoceptive signals. Within this framework, the interoceptive system minimizes free-energy, or the discrepancy between predictions and interoceptive signals. Free-energy can be minimized either by updating predictions about the causes of the sensory signals (perceptual updating), or by acting to change autonomic states such that bodily states are more predictable (active inference).

The free-energy principle is currently in vogue in neuroscience. We are no longer strangers to the idea that perception is an active iterative process between abstract representations (predictions) and sensory feedback (prediction errors) (Clark, 2013). The basic idea of PC in the cognitive sciences began with the notion of neural energy (Helmholtz, 1860) and it has been present since in the form of theoretical proposals and empirical findings, especially in the visual domain (Lee and Mumford, 2003). Therefore Seth's proposal that sensory processing involves predictions is nothing new. What is new in Seth's model is that perception of internal body signals (interoception), paralleling the perception of external signals, relies on top-down predictions of the causes of the sensory input, rather than being a passive, bottom-up process.

Is then Seth's interoceptive inference model an interesting proposal to explain emotion awareness and selfhood? My opinion is yes and that it is worth investigating. However, there are some aspects to consider before designing studies to empirically test Seth's model.

Seth's model builds on three main assumptions. First, emotions are defined as affective states relying on interactions between top-down interoceptive predictions and bottom-up interoceptive prediction errors. Following the principles of PC, there is a constant attempt to minimize the discrepancy between the predicted and the actual sensory events, either through updating perceptual expectations or through active inference (Friston et al., 2010). As Seth nicely explains, active inference in interoception occurs when predictions are transcribed into reference points that trigger autonomic homeostatic regulation, occurring when the weight of the error is low and attention to errors is attenuated (Gu et al., 2013).

Fortunately, advances on biomedical tools allow us to experimentally monitor the body's physiological signals. Although, some methodological challenges still remain when investigating interoception. This general issue may also impact on PC studies of interoception. However, applying PC to interoception, as proposed in Seth's model, may allow us to overcome these challenges. The main argument of PC is that all sensory systems are linked by working under identical code schemes (Friston and Kiebel, 2009). Therefore, Seth's PC model allows us to apply knowledge from visual and other domains to investigate brain and behavioral mechanisms of interoception. Neuroimaging studies have demonstrated direct evidence of PC in visual brain areas (Egner et al., 2010; Wyart et al., 2012). Likewise, Seth's anatomical predictions (i.e., anterior insular cortex -AIC) can be tested by using multivoxel pattern analysis approaches, in combination with orthogonal experimental designs where the stimulus presentation probability is held constant in all conditions (Egner et al., 2010).

The second assumption in Seth's model refers to the AIC as the key structure that generates, compares, and updates interoceptive predictions. Empirical evidence has shown that AIC houses a secondary associative area where interoceptive, exteroceptive, and motivational signals converge (Seth and Critchley, 2013). An important principle of PC explains that the surprisal generated in one unimodal system can be explained away by inferences in other system via high-order neural areas (Apps and Tsakiris, in press). Considering the multimodal nature of the AIC, one could suggest that the errors in the interoceptive signal can be explained by exteroceptive inferences (or vice versa) and that the interoceptive generative models are only a part of the way the system explains errors. Whether the AIC exclusively codes the surprisal evoked by interoceptive signals or, alternatively, if the AIC is involved in top-down general predictions directed to a more specialized interoceptive circuit, still remain open questions.

The third crucial aspect of Seth's model is the concept of selfhood. Seth has employed the idea that selfhood is formed by the integration of predictive interoceptive and exteroceptive signals (Tajadura-Jimenez and Tsakiris, in press). Individual differences in the accuracy of interoceptive awareness influence integration of interoceptive and exteroceptive information, as shown by studies in body illusions (Tsakiris et al., 2011). Individuals with low accuracy show more susceptibility to body illusions, which Seth interprets as lower precision-weighting of interoceptive prediction errors. However, although a free-energy model of self has been proposed (Apps and Tsakiris, in press), as yet there is no evidence to suggest that self-processing follows the principles of PC.

Another crucial factor that may influence interoceptive awareness, and therefore self-awareness, is attention. In PC, attention is considered to be a mechanism that optimizes the precision of prediction errors during hierarchical inference (Feldman and Friston, 2010). For example, studies in vision have demonstrated that attention enhances the neural specificity for expected vs. unexpected stimuli in visual cortex (Jiang et al., 2013). Similarly, directing attention toward internal body signals might increase the precision of interoceptive prediction errors and therefore improve interoceptive awareness. An individual's attention to the body can be significantly enhanced by the practice of Mindfulness (Farb et al., 2013), which also has the effect of enhancing both cortical responses of interoceptive attention and self-reported interoceptive awareness (Mehling et al., 2013). Within Seth's model this might increase the accuracy of interoceptive inference, emotions, and self-awareness.

Therefore, I agree with Seth's proposal that the brain is a prediction machine that integrates interoceptive and exteroceptive information in a Bayesian way. However, future research is needed to elucidate the internal properties of the interoceptive inference.
